# Reduction of relative resting myocardial blood flow is related to myocardial delayed enhancement, T2-signal abnormalities, left-ventricular wall thickness and age in patients with hypertrophic cardiomyopathy

**DOI:** 10.1186/1532-429X-14-S1-P158

**Published:** 2012-02-01

**Authors:** Katja Hueper, Antonia Zapf, Jan Skrok, Aurelio C Pinheiro, Thomas A Goldstein, Jie Zheng, Stefan L Zimmerman, Ihab R Kamel, M Roselle Abraham, Frank Wacker, David A Bluemke, Theodore Abraham, Jens Vogel-Claussen

**Affiliations:** 1 Radiology, Hannover Medical School, Hannover, Germany; 2 Radiology, Johns Hopkins University, Baltimore, MD, USA; 3 Division of Cardiology, Johns Hopkins University, Baltimore, MD, USA; 4 Institute for Biometry, Hannover Medical School, Hannover, Germany; 5 Radiology, Washington University School of Medicine, St Louis, MO, USA; 6 Department of Mathematics, University of California, Los Angeles, Los Angeles, CA, USA; 7 National Institutes of Health, Department of Radiology and Imaging Sciences, Bethesda, MD, USA

## Summary

By cardiac MRI we investigated the relationship of resting myocardial blood flow (MBF) in hypertrophic cardiomyopathy (HCM) patients to important parameters of disease. MBF was quantified on pixel-by-pixel basis in 804 segments in 70 patients. Relative MBF negatively correlated with left-ventricular (LV) wall thickness (p<0.001), extent of myocardial delayed enhancement (MDE; p<0.001), edema (T2 bright; p<0.001), T2 dark signal (p<0.001) and age (p=0.032), but not LV outflow gradient (p=0.901). Different perfusion patterns in segments with reduced and elevated resting perfusion were observed, which may represent different stages of HCM.

## Background

Myocardial perfusion abnormalities are common in patients with hypertrophic cardiomyopathy (HCM) and seem important for patient prognosis. The purpose of this study was to quantify the distribution of resting left ventricular (LV) myocardial blood flow (MBF) by MRI and to determine its relationship to important parameters of disease such as LV wall thickness, myocardial delayed enhancement (MDE), T2-signal abnormalities, LV outflow tract obstruction and age.

## Methods

Seventy patients (mean age 52 years) with HCM underwent cardiac MRI (1.5 T system, Avanto, Siemens Health Care). For perfusion imaging a bolus of gadopentetate dimeglumine was injected at a dose of 0.04 mmol/kg bodyweight. Two short axis views at the basal and mid-ventricular level were acquired using a saturation preparation SSFP sequence. Cine, T2-weighted dark blood turbo spin echo and MDE images were obtained in the short axis view, matching the slice location of the cardiac perfusion sequence. The LV myocardium was divided into 6 segments in each short-axis slice. Extent of MDE and the presence of T2 signal abnormalities were scored semiquantitatively for each myocardial segment: Edema and T2 dark signal, defined as focal increase or respectively decrease of T2-signal when compared to remote T2-signal of the myocardium, were scored as absent or present. MDE was scored from 0 to 4 based on the percentage area with hyperenhancement. Quantitative MBF maps were generated on a pixel-by-pixel basis using the Fermi function model. Relative perfusion was calculated for each myocardial segment by referring to segments with an LV wall thickness <20mm and MDE ≤25% in the same patient. Simple and multiple linear regression models with repeated measures were used to determine the association of relative resting MBF with LV wall thickness, MDE score, T2 bright signal score, T2 dark signal score, LV outflow gradient and age. Furthermore, T2-signal changes and MDE patterns were described in myocardial segments with elevated and reduced perfusion.

## Results

**Figure 1:**
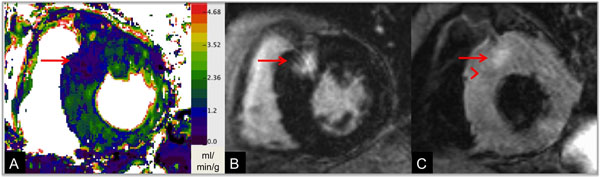
70-year-old patient with HCM and an LV outflow gradient at rest of 28 mmHg. Intense, well-defined MDE in the septum at the anterior right ventricular insertion (B) with corresponding hypo-perfusion (relative MBF = 56%, A). T2-weighted imaging depicts high signal, indicating edema (arrow), and adjacent low signal (arrow head, C).

804 segments in 70 patients were included in the analysis. In a simple linear regression model LV wall thickness (p<0.001), extent of MDE (p<0.001), T2 bright signal (p<0.001), T2 dark signal (p<0.001) and age (p=0.032) negatively correlated with relative resting MBF. The LV outflow gradient did not show an effect on resting perfusion (p=0.901). Multiple linear regression analysis revealed that MDE (p<0.001), T2 bright signal (p=0.026) and T2 dark signal (p=0.019) were independent predictors of relative resting MBF. Different MDE patterns in segments with reduced and elevated resting perfusion were observed (Fig 1).

## Conclusions

In HCM resting MBF is significantly reduced depending on LV wall thickness, extent of MDE, focal T2 bright (edema) and dark (confluent/chronic fibrosis) signal changes and age. Different myocardial resting perfusion patterns could be defined, which may represent different stages of disease.

## Funding

None.

